# Characterization and Genomics of Pectinolytic Bacteria Isolated from Soft Rot Symptomatic Produce

**DOI:** 10.3390/pathogens13121096

**Published:** 2024-12-12

**Authors:** Kyla Radke, Brandon Rivers, Mya Simpkins, Jacob Hardy, Jeffrey K. Schachterle

**Affiliations:** Department of Microbiology and Molecular Biology, Brigham Young University, Provo, UT 84602, USA; kr539@byu.edu (K.R.);

**Keywords:** soft rot, pectinolytic, *Pectobacterium*, *Pseudomonas*, *Chryseobacterium*

## Abstract

Bacterial soft rot causes major crop losses annually and can be caused by several species from multiple genera. These bacteria have a broad host range and often infect produce through contact with soil. The main genera causing bacterial soft rot are *Pectobacterium* and *Dickeya*, both of which have widespread geographical distribution. Because of many recent renaming and reclassifications of bacteria causing soft rot, identification and characterization of the causative agents can be challenging. In this work, we surveyed commercially available produce exhibiting typical soft rot symptoms, isolating pectinolytic bacteria and characterizing them genetically and phenotypically. We found that in our sampling, many samples were from the genus *Pectobacterium*; however, other genera were also capable of eliciting symptoms in potatoes, including an isolate from the genus *Chryseobacterium*. Genomic analyses revealed that many of the *Pectobacterium* isolates collected share prophages not found in other soft rot species, suggesting a potential role for these prophages in the evolution or fitness of these isolates. Our *Chryseobacterium* isolate was most similar to *C. scophthalmum*, a fish pathogen, suggesting that this isolate may be a crossover pathogen.

## 1. Introduction

Bacterial soft rot is a disease complex that causes severe crop loss worldwide. Multiple bacterial genera cause this disease, with the genera *Pectobacterium* and *Dickeya* being among the most common [1]. These pathogens leave necrotic spots on the soft tissues of many plants, such as stems, leaves, tubers, and flesh, of a wide variety of cultivated vegetables. Soft rot is usually observed as the gradual presence of water-soaked lesions where the tissue becomes depressed, soft, mushy, slimy, and discolored [2]. This results in significant yield losses, affecting both the pre-harvest period and the post-harvest period due to latent infection [3]. Soft rot can occur over a wide temperature range, with most pathogens infecting optimally in the range of 21–27 °C, and is most severe when oxygen is limited [4]. Crops in storage, such as potato tubers, may have restricted access to oxygen when stacked on top of each other with limited airflow [5]. *Pectobacterium* and *Dickeya* survive in the soil and on the surface of crops, infecting plants through natural openings and wound sites [6]. Currently, there are no effective treatments once soft rot has infected plant tissues.

Bacteria of the *Pectobacteriaceae* family release a variety of pectinases as exoenzymes and endoenzymes for pectin degradation [7]. These enzymes break down pectin, a polysaccharide present in the middle lamella of plant cell walls, allowing access to a nutrient-rich environment [8]. Pectinases are classified into hydrolases, esterases, and lyases based on their active sites. Hydrolases, also known as polygalacturonases (PGs), break down pectic substances by catalyzing the hydrolysis of α-1,4-glycosidic bonds between galacturonic monomers that form chains in the pectin molecule. Esterases, also known as pectin methylesterases (PMEs), remove the acetyl and methoxyl groups from pectin. Lyases catalyze the cleavage of glycosidic bonds through β elimination mechanisms [9]. These enzymes have been isolated and used for industrial applications [10]. When *Pectobacteriaceae* bacteria start to infect the plant and grow quickly in numbers, an overwhelming amount of pectinase enzymes are produced, which results in soft rot. Pectin binds plant cells together, thus causing plant structures to fall apart into mushy, discolored, and sunken pits when infected. Members of the *Pectobacterium* and *Dickeya* genera are rod-shaped, facultative anaerobes and are motile by means of peritrichous flagella [11].

Although *Pectobacterium* and *Dickeya* are perhaps the most established genera associated with soft rot [12], a variety of other species have been implicated, including *Pseudomonas*, *Bacillus*, and *Clostridium*. *Pseudomonas*, a large and diverse group of bacteria [13], has been identified through 16S rDNA sequencing as a causative agent of soft rot in potatoes [14]. The versatile, spore-forming genus *Bacillus* has also exhibited pathogenicity on apples, pears, and other produce [15]. However, it has also been proposed as a potential biological control method against the soft rot pathogen *Pectobacterium carotovorum* [6]. *Clostridium*, a spore-forming, primarily anaerobic genus, has been isolated from symptomatic sweet potatoes and other crops [16].

As classification techniques have become more sophisticated and accurate, methods of genome sequencing, such as whole-genome sequencing, have developed, leading to the necessity of the reclassification of previously identified species, including those causing soft rot [17]. For example, the once broad genera *Erwinia* became divided into subcategories *Pectobacterium* and *Dickeya* through 16s rRNA comparative analysis [17,18]. This is a critical differentiation in the field of soft rot and plant disease because *Pectobacterium* and *Dickeya* are distinct in their host range and pathogenicity, making proper classification crucial for studying and understanding the virulence mechanisms of pathogenic bacteria. Sophisticated classification efforts will almost certainly yield the identification of novel and previously unknown pathogenic bacteria contributing to soft rot [19]. However, as more novel bacteria are identified, the demand for increasingly advanced molecular techniques will rise to ensure accurate and rapid identification. Technology that is more expensive and less widely available will potentially be needed to make precise classifications between isolates of high similarity, complicating soft rot research endeavors in lower-resource areas and certain laboratories.

Additionally, there may be regulatory concerns implicated with advancements in diagnostic technology, especially in the field of agriculture. In August 2019, the U.S. Department of Agriculture ceased requiring permits for the interstate transfer of *Pectobacterium carotovorum* strains originating from within the continental United States (Federal Register #2019-13246). However, two months later, a novel species, *P. versatile*, was formed from isolates formerly considered *P. carotovorum* [20]. Although the ongoing refinement and revision of pathogen taxonomy can create improved classification and understanding of pathogen behavior, it can also foster confusion around regulatory requirements for newly formed groups. Rapidly changing taxonomy also creates differences in the reporting of diagnostic results from different laboratories, which limits the understanding of which pathogens are present in specific geographic areas.

In our study, we surveyed pectinolytic bacteria in commercial produce and characterized the isolates obtained via both genotypic and phenotypic methods. As expected, we identified *Pectobacterium* species; however, we also found isolates from several other genera, including isolates pathogenic to potatoes that represent potentially novel species. We further found an isolate of *Chryseobacterium scophthalmum*, closely related to fish pathogens [21,22], which may represent a crossover pathogen. While the *Pectobacterium* isolates were highly similar, phenotypic differences and distinct prophage profiles revealed differences between strains. Our survey advances our understanding of the pathogens currently causing soft rot in consumer-available produce and provides a foundation for further work into the mechanisms that make these pathogens successful.

## 2. Materials and Methods

### 2.1. Culture and Isolation Conditions

Unless otherwise specified, bacterial isolates were routinely cultured at 30 °C in LB medium (10 g/L tryptone, 5 g/L NaCl, 5 g/L yeast extract; for solid media: 15 g/L agar). Produce samples used in isolations were selected from commercially acquired produce and exhibited symptoms commonly associated with bacterial soft rot, primarily water-soaked tissues and sunken lesions. Samples used for isolation were all collected in 2024 and are specified in Supplemental Table S1. Because the produce was commercially obtained from consumer grocery stores, data regarding the location of cultivation or the specific varieties of plants are unknown.

Original isolations of bacteria from symptomatic plant tissues were conducted using crystal violet pectate medium (CVPM) [23]. Briefly, an inoculation loop was used to scrape water-soaked tissue from the plant sample and streaked for isolation on CVPM plates. Colonies forming sunken pits on CVPM were re-isolated using CVPM until pure cultures were obtained.

Genetic identification of the bacteria was conducted via Sanger sequencing of the 16S rDNA gene. Briefly, a fragment of the 16S rDNA gene was PCR-amplified using primers 27F and 1492R [24]. PCR fragments were purified using ethanol precipitation and digestion with exonuclease I and alkaline phosphatase (New England Biolabs, Ipswich, MA, USA). Sanger sequencing reactions and capillary electrophoresis of purified DNA fragments were conducted by the BYU DNA Sequencing Center (Provo, UT, USA) with either the 27F or 1492R primers. A BLAST [25] of the obtained sequences against the NCBI non-redundant database was used to determine the genus and, when possible, the predicted species of the isolate, taking the top BLAST hit as the putative identity of the isolate.

### 2.2. Phenotypic Characterizations

All pectinolytic isolates were tested using the following phenotypic tests: pathogenicity to potato and Chinese (Napa) cabbage, swimming motility, swarming motility, production of acyl homoserine lactones (AHLs), lactose fermentation, and protease production.

#### 2.2.1. Pathogenicity Assays

For potato pathogenicity, 1 cm slices of russet potatoes were prepared and placed in sterile petri dishes. Potato inoculum was prepared by growing each isolate overnight in LB and normalizing the culture density to OD600 = 0.4. A 2 µL droplet (~2 × 10^6^ CFU) was inoculated on the center of each potato slice. For Chinese (Napa) cabbage pathogenicity, cabbage leaves were placed in plastic totes cleaned with 70% ethanol. For cabbage inoculations, bacterial isolates were grown for 24 h on LB agar, and single colonies were picked with a pipette tip and inoculated by stabbing the tip 2 mm into the surface of the cabbage leaf. Inoculated produce was incubated at 30 °C and evaluated for symptom development every 24 h for 3 days. Symptoms included water soaking, tissue maceration, and discoloration. Potato slices were photographed and analyzed using ImageJ [26] to determine the surface area covered by disease 48 h post-inoculation.

#### 2.2.2. Swimming and Swarming Motility

Swimming and swarming motility assays were conducted as described previously [27]. In short, soft agar plates containing 0.25% agar (swim) or 0.3% agar (swarm) were prepared. Swim plates were inoculated by stab inoculation, and swarm plates were surface inoculated with 3 µL of overnight culture. Plates were photographed 24 h post-inoculation, and the area covered by motile bacteria was quantified using ImageJ [26].

#### 2.2.3. AHL Production

Acyl homoserine lactone production was assessed as described [28,29] using the *Agrobacterium fabrum* reporter strain NTL4 pZLR4. Briefly, soft agar plates containing the *A. fabrum* reporter as well as X-gal (5-bromo-4-chloro-3-indolyl-beta-D-galactopyranoside) were prepared. A 3 µL droplet of overnight culture of each isolate was dripped onto a plate containing the reporter strain and incubated overnight. A positive reaction was evidenced by the production of a blue color in the soft agar surrounding the test strain.

#### 2.2.4. Lactose Fermentation

LB agar medium was prepared to contain 1% (*w*/*v*) lactose and phenol red dye as a pH indicator. At neutral pH, the media is a bright red color. When isolates were grown on this media, a color change from red to yellow was interpreted as a lowering of the pH in the media around colonies fermenting lactose.

#### 2.2.5. Extracellular Protease Activity

LB agar containing 1% (*w*/*v*) skim milk powder was prepared, and isolates were cultured in this medium. A halo of clearing around colonies was taken as evidence of extracellular protease activity.

#### 2.2.6. Biofilm Formation

Biofilm formation by each isolate was assayed using 96-well plates as described previously [30]. Briefly, isolates were grown overnight in LB broth. A 5 µL volume of overnight culture, standardized to an absorbance of 0.4, was added to 100 µL of LB in the well of a 96-well plate, and the plate was incubated at 30 °C for 48 h. Following incubation, media and planktonic cells were removed and plates were allowed to dry. Adherent cells were stained with 100 µL of 1% crystal violet, and cells were washed three times with deionized water. The dye was solubilized using 70% ethanol, and the absorbance of the solubilized dye was measured at 595 nm.

### 2.3. Genome Sequencing

For genome sequencing, select bacterial isolates were cultured in LB medium overnight at 30 °C. Genomic DNA was isolated from 5 mL of culture using the Quick DNA Miniprep Plus Kit (Zymo Research, Irvine, CA, USA) according to the manufacturer’s instructions. Purified DNA sample concentration and quality were verified spectrophotometrically and by agarose gel electrophoresis. Samples were submitted to Plasmidsaurus (Eugene, OR, USA) for sequencing using long-read sequencing and assembly approach. All genome sequences generated are available on NCBI under BioProject number PRJNA1183984.

### 2.4. Computational Analyses

Reference genomes of all type strains from the following genera were obtained from the NCBI GenBank database: *Pectobacterium*, *Dickeya*, *Pseudomonas*, and *Chryseobacterium*. Where type strains were used, all GenBank genomes represent taxonomically named species. The average nucleotide identity between these type strains and our isolates with sequenced genomes was calculated using fastANI [31]. Further comparisons were made using additional genomes from the species *Pectobacterium carotovorum* and *Pectobacterium versatile.* Whole genome alignments were generated and visualized using the progressiveMauve aligner [32] with default parameters.

Lysogenic bacteriophage within the sequenced *Pectobacterium* genomes were identified using Phastest [33]. Extracted sequences of identified lysogenic bacteriophages were further analyzed using fastANI [31]. The Newick tree was generated with ETEToolkit [34] for visualization.

## 3. Results

### 3.1. Isolation and Identification

We collected commercial produce samples exhibiting typical soft rot symptoms and plated them onto CVPM to isolate pectinolytic bacteria associated with these samples (Figure S1). From 47 produce samples, we collected 28 isolates of pectinolytic bacteria (Table S1). When apparently distinct isolates were obtained from a single sample, these isolates were designated ‘a’ or ‘b’ isolates. We used 16S rDNA sequencing to identify the genus and species of the pectinolytic isolates, when possible, and found isolates from nine different genera: *Achromobacter*, *Chryseobacterium*, *Escherichia*, *Lelliottia*, *Pantoea*, *Pectobacterium*, *Pseudomonas*, *Sphingobacterium*, and *Stenotrophomonas* (Table 1 and Table S2).

### 3.2. Phenotypic Characterization

**Figure 1:**
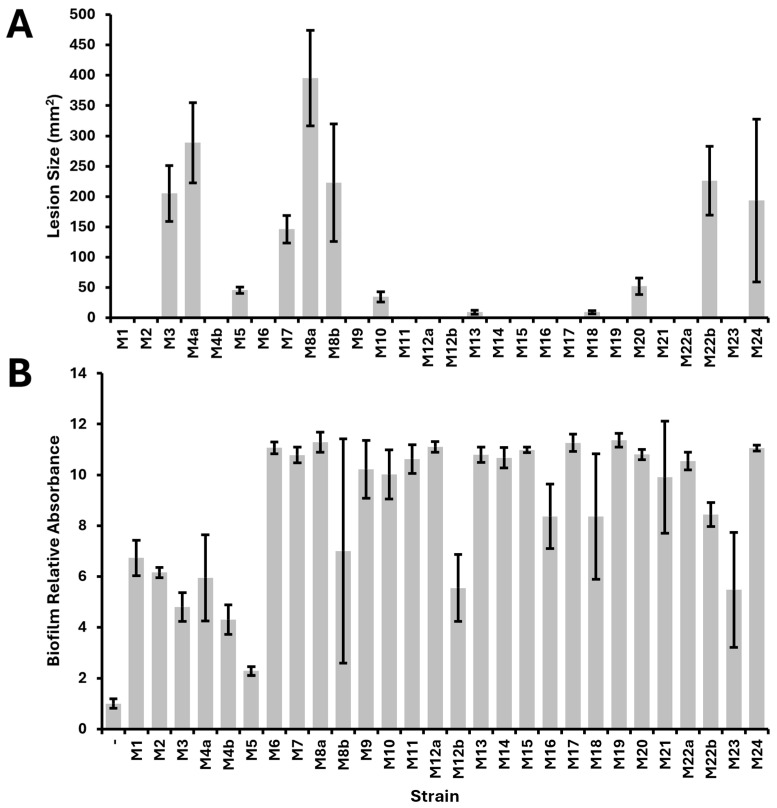
Virulence of isolates as measured by area of lesion on inoculated potato slices (**A**) and biofilm formation in 96-well plates following static growth for 48 h (**B**).

Because each of our isolates was collected from plant tissue exhibiting soft rot symptoms, we tested each of the isolates against potato and Chinese (napa) cabbage to determine whether these isolates were pathogenic to these hosts (Figures S2 and S3). We found that only 12 of the 28 isolates (43%) were pathogenic to potatoes, and only three (11%) were pathogenic to Chinese (napa) cabbage (Table 1). The *Pectobacterium* isolates, which were mainly isolated from different hosts, were all pathogenic to potatoes as they caused soft rot symptoms as they grew on the potatoes. We observed a high degree of variability in the size of lesions caused by our different isolates when used to inoculate potato slices (Figure 1A).

We assessed whether each of the 28 isolates exhibited various phenotypes associated with virulence, including swimming and swarming motility, biofilm formation, production of acyl-homoserine lactones, fermentation of lactose, and extracellular protease activity (Table 1). All of the isolates exhibited both swimming and swarming motility. All of the isolates appeared to form biofilms, with crystal violet staining higher than the negative control (Figure 1B). Several strains appeared to form strong biofilms with staining 10-fold higher than the negative control; however, strain M5 exhibited the lowest biofilm formation of all the isolates, at only about twice the crystal violet staining of the negative control. Nine of the isolates (32%) produced acyl homoserine lactones detectable with our *A. fabrum* reporter system [28,29]. Twelve of the isolates (43%) fermented lactose, and 24 of the isolates (86%) exhibited extracellular protease activity.

We used linear regression and computed F-statistics for pairwise correlation between the pathogenicity-to-potato trait and the other virulence-associated phenotypic traits we tested. The additional traits included motility, lactose fermentation, production of acyl homoserine lactones, and extracellular protease activity. We found a significant pairwise correlation (*p* < 0.001) between the production of acyl homoserine lactones and pathogenicity to potatoes. The correlations for all other virulence traits were not statistically significant.

### 3.3. Genome Sequencing and Assemblies

We selected a subset of 11 isolates obtained in our survey for whole genome sequencing, with a focus on isolates pathogenic to potatoes. The isolates sequenced and sequencing results are summarized in Table 2. The average sequencing depth was 76× across all samples. All of the *Pectobacterium* and the *Chryseobacterium* genomes were assembled to a single contig with no plasmids. The *Pectobacterium* isolates appeared to have the smallest genome size, ranging between 4.57 Mb and 5.14 Mb, while the *Pseudomonas* isolates appeared to have larger genome sizes, ranging between 6.27 Mb and 6.93 Mb. All of the *Pseudomonas* genomes sequenced had at least one plasmid present, suggesting that their genomes may be more complex.

### 3.4. Genome Alignments

**Figure 2:**
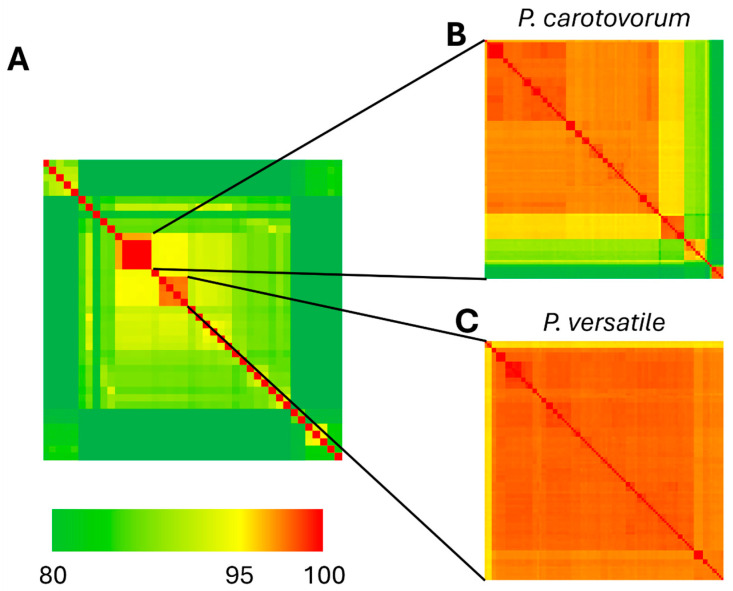
Average nucleotide identity of *Pectobacterium* isolates represented as a heatmap. (**A**) ANI across all type strains of *Pectobacterium* and *Dickeya* species in RefSeq database (*n* = 41 genomes). (**B**) ANI across all genomes entered in RefSeq database as *P. carotovorum* (*n* = 103 genomes). (**C**) ANI across all genomes entered in RefSeq database as *P. versatile* (*n* = 105 genomes).

We compared the genomes sequenced in our study with genomes available in the RefSeq database by calculating average nucleotide identity (ANI). We first compared our *Pectobacterium* genomes to the 35 type strain genomes available from the genera *Pectobacterium* and *Dickeya*, which confirmed that isolates M3, M4a, M8a, and M8b are *P. carotovorum* and isolates M10 and M22b are *P. versatile* (Figure 2A, Table S3). We then calculated ANI across M3, M4a, M8a, M8b, and all 99 available *P. carotovorum* genomes (Figure 2B, Table S4), which indicated that our isolates formed their own highly related cluster, more similar to each other than any other *P. carotovorum* genome. We similarly calculated ANI for M10, M22b, and all 103 available *P. versatile* genomes (Figure 2C, Table S5) and found M10 and M22b to be closely related to *P. versatile* strains isolated from cabbage, potato, and surface water, and from geographically diverse areas across the United States and Europe.

**Figure 3:**
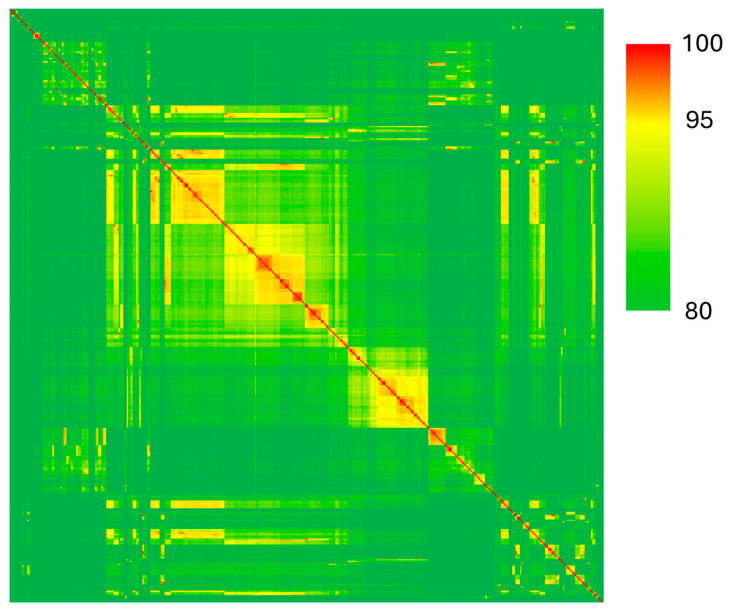
Average nucleotide identity of *Pseudomonas* isolates from our survey and type strains of all species in the RefSeq database (*n* = 375 genomes).

For our four genomes from the genera *Pseudomonas*, we calculated ANI to compare these isolates to the 369 type strain genomes available (Figure 3, Table S6). This analysis confirmed that strain M12a is *Pseudomonas alloputida* (ANI = 96.71%), M13 is *Pseudomonas marginalis* (ANI = 99.22%), and M14 is *Pseudomonas petroselini* (ANI = 98.81%). The highest ANI found for strain M20 was for *Pseudomonas tensinigenes* (ANI = 93.27%), a member of the *P. koreensis* group.

**Figure 4:**
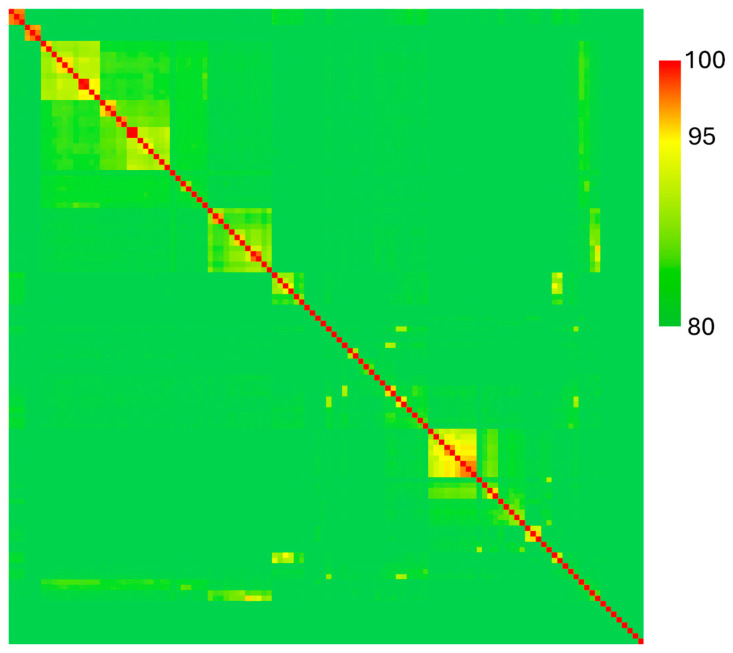
Average nucleotide identity of the *Chryseobacterium* isolate and all type strains from *Chryseobacterium* species in the RefSeq database (*n* = 118 genomes).

We also calculated ANI for our *Chryseobacterium* isolate, M5, and the 117 type strain genomes available for this genus (Figure 4, Table S7). We found that isolate M5 is similar to *Chryseobacterium scophthalmum* (ANI = 95.31%), which is a fish pathogen.

**Figure 5:**
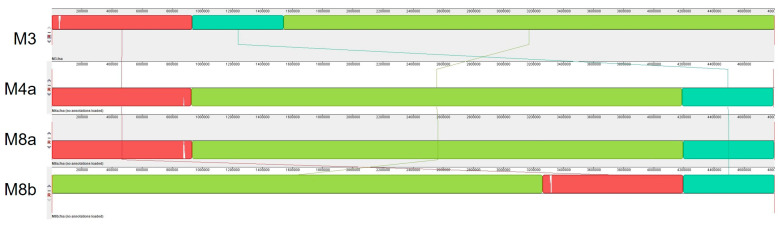
Whole genome alignments of *P. carotovorum* isolates M3, M4a, M8a, and M8b. Alignment and visualization generated by Mauve.

Because we found that average nucleotide identity had little power to resolve differences between our closely related *Pectobacterium carotovorum* isolates, we sought to better understand the relationships between these genomes and isolates. We first aligned the full-length genomes of these isolates and found that strains M3 and M8b had large rearrangements, but strains M4a and M8a shared the same structure (Figure 5).

**Figure 6:**
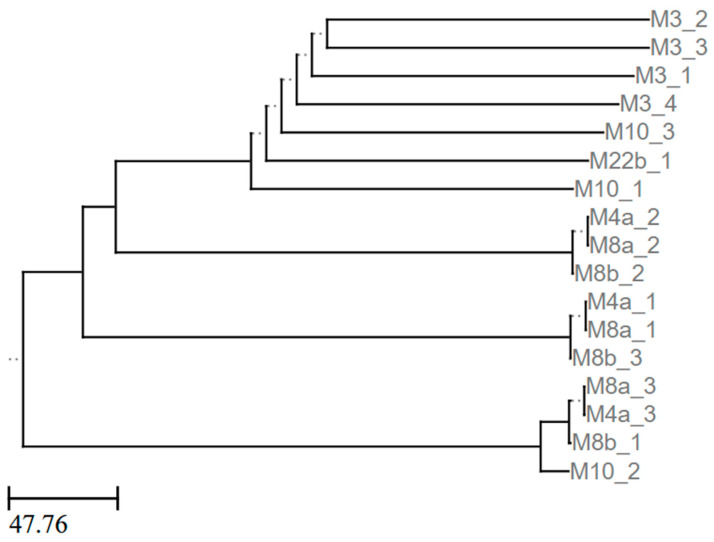
Prophage dendrogram. Newick tree generated by ANIclustermap using sequences of prophages predicted by Phastest in newly sequenced *Pectobacterium* genomes. Representation rendered by ETEToolkit.

To test whether the *Pectobacterium* strains also shared horizontally acquired genetic elements or if unique DNA fragments could serve to separate these strains, we computationally predicted prophage elements in each of the *Pectobacterium* genomes we sequenced (Table S8) and then compared the sequences of the predicted prophages (Figure 6). We found that all of the genomes contained at least one predicted prophage. We further found that although some isolates, such as M4a and M8a, shared their predicted prophages, other isolates, such as M3, carried almost entirely distinct prophages from their otherwise genomically close relatives. Top BLAST hits for each of the predicted prophages show that in addition to *P. carotovorum* and *P. versatile*, similar prophages are found in *P. brasiliense*, *P. parvum*, *P. odoriferum*, and *P. wasabiae* (Table S9).

## 4. Discussion

In this study, we isolated 28 pectinolytic bacteria from various produce samples exhibiting typical bacterial soft rot symptoms. These isolates came from nine different genera, but only isolates from three of these genera, *Chryseobacterium*, *Pectobacterium*, and *Pseudomonas*, were pathogenic to potatoes. Only isolates from *Pectobacterium* elicited disease symptoms on Chinese (Napa) cabbage. Although several species from the genera *Pectobacterium* and *Dickeya* are typically associated with bacterial soft rot, in our survey, we only obtained isolates from *P. carotovorum* and *P. versatile*. *P. versatile* represents a group of strains that was recently elevated to its own species and was formerly classified with *P. carotovorum* [20]. Our genomic observations that *P. versatile* and *P. carotovorum* have average nucleotide identity below 96% align with their division into two species [20]. Furthermore, our two *P. versatile* isolates, M10 and M22b, both infect Chinese (Napa) cabbage, but strain M3 was the only one of four *P. carotovorum* isolates to infect this host. Interestingly, strains M3, M10, and M22b each carry prophages in their genome distinct from the other *Pectobacterium* isolates and their predicted prophages. BLAST searches showed that nine of the predicted prophages are most similar to prophages found in *P. carotovorum* and *P. versatile*, while the remaining prophages are more similar to prophages from *P. brasiliense*, *P. odoriferum*, *P. parvum*, and *P. wasabiae*. Interestingly, one cluster of phages (M4a_2, M8a_2, and M8b_2) only had matches along 28% of the length of the prophage sequence. Another five phages had matches less than 80% of the length of the sequence. Future work is needed to address the underlying mechanisms of host specificity and the potential roles of horizontally acquired genetic elements, such as phage, in contributing to host range.

Phenotypic characterization of our isolates showed that many of them exhibited traits often associated with virulence in pathogens. Although the traits assessed are considered virulence factors for several pathogens, only acyl homoserine lactone production was significantly associated with pathogenicity in potatoes. All isolates exhibited swimming and swarming motility and most expressed extracellular protease activity. Because our isolates came from diverse host plants and represent various taxa, it is unclear whether these isolates use these phenotypic traits for virulence in other host plants but do not include potatoes as hosts. Thus, we hypothesize that the significant correlation between acyl homoserine lactone production and potato pathogenicity is because most of the potato pathogenic isolates are *Pectobacterium* isolates and all of the *Pectobacterium* isolates produce acyl homoserine lactones.

All of the *Pectobacterium* isolates, both *P. carotovorum* (M3, M4a, M8a, M8b) and *P. versatile* (M10, M22b), exhibited all measured phenotypic characteristics, with the exception of M10, which did not display protease activity. While strain M10 was pathogenic to potatoes, the lesion size caused by M10 was less than 50 mm^2^, whereas the lesions caused by the other *Pectobacterium* isolates were closer to 200 mm^2^. Additional work will reveal whether the reduced lesion size is directly or indirectly linked to the lack of extracellular protease activity in strain M10, as extracellular proteases are known to be important for full virulence in some plant-pathogenic bacteria but dispensable for others [35,36,37]. More broadly, there was no significant correlation between protease activity and pathogenicity to potatoes among all of our isolates.

Most of the *Pseudomonas* isolates we obtained were not pathogenic to potatoes, except for isolates M13, M18, and M20. Based on our average nucleotide identity analyses, strain M13 is a new isolate of *Pseudomonas marginalis*, a species that has previously been associated with soft rot disease [14,38]. For strain M20, the highest average nucleotide identity observed with another genome was with *Pseudomonas tensinigenes*, a member of the *P. koreensis* group [39]. However, the average nucleotide identity between these strains was only 93.27%, well below the typical species-level thresholds of 95–96%. We hypothesize that M20 represents a novel species that is capable of causing soft rot disease in potatoes and potentially other diverse hosts, as it was isolated from spinach. On potatoes, the lesion size caused by M20 was comparable to that of the *Pectobacterium* isolate M10. It is recognized that various species of *Pseudomonas* can cause soft rot, and the species most commonly associated therewith are *P. glycinae*, *P. cichorii*, *P. marginalis*, and *P. viridiflava* [38,40,41,42]. Of these species, we recovered one isolate of *P. marginalis* (M13) and a putatively novel species associated with soft rot (M20).

The genus *Chryseobacterium* is diverse, and its members occupy several environmental niches [43]. Many *Chryseobacteria* are not pathogenic to humans and animals, and of those that are, many tend to be weakly pathogenic, such as *C. indologenes*, formerly *Flavobacterium indologenes* [44]. *C. indologenes* has also been reported as a plant pathogen, causing root rot in Panax ginger [45]. On the other hand, *Chryseobacterium* isolates have been reported to exhibit plant-beneficial effects [46,47]. Some *Chryseobacterium* isolates also exhibit the ability to digest complex organic molecules, such as herbicides [48]. Our *Chryseobacterium* strain M5 contains predicted pectate lyase (ACI513_RS09935) and pectin esterase (ACI513_RS09930) genes located adjacent to each other in the genome, supporting a role for this strain in host cell wall degradation. Other strains of *Chryseobacterium* have previously been reported to express pectinolytic activity [10,49]. By our calculations, *Chryseobacterium* strain M5 was most similar to *Chryseobacterium scophthalmum* based on average nucleotide identity. *C. scophthalmum* is pathogenic to fish, being originally isolated from turbots in the Atlantic Ocean [21,22]. Because a fish pathogen is the closest relation to M5, this isolate may represent a crossover pathogen, evolving from an animal to a plant pathogen. It will be interesting to determine in future work whether this particular isolate also exhibits any animal pathogenic activity. However, because the average nucleotide identity between M5 and *C. scophthalmum* is 95.3%, we hypothesize that M5 may represent a novel species of *Chryseobacterium*. Further work to understand the evolution, host range, and virulence of this isolate and its relatives is warranted.

Among the other isolates obtained, both *Stenotrophomonas* isolates (M15 and M19), which were obtained from different hosts, displayed the same phenotypic characteristics and were not pathogenic to potatoes. Neither of the isolates that came from symptomatic potatoes (M15 and M16) were pathogenic to potatoes under our experimental conditions and time frame. Our isolation approach used crystal violet pectate medium to isolate from tissues. Because of the crystal violet component of the medium, it was anticipated that Gram-positive pectinolytic bacteria would not be isolated in this approach. Indeed, our isolations only yielded bacteria from Gram-negative taxonomic groups. We successfully isolated pectinolytic bacteria from more than half of our plant samples and may have found additional isolates had our methods also targeted Gram-positive pectinolytic bacteria; thus, some of our pectinolytic isolates may have been non-pathogenic because they are members of disease complexes in which multiple diverse bacteria work together to cause soft rot.

Altogether, our work demonstrates that there are abundant and diverse bacteria associated with and causing post-harvest soft rot disease in commercial produce, including a potential crossover pathogen. It is important to be aware of this diversity in future work to develop and improve disease control methods. Our survey work and the genomic resources we have developed lay an initial foundation for work on these pathogens to better understand their virulence mechanisms and host specificities. Furthermore, a lack of redundancy in the isolates we collected from our survey indicates that there is value in ongoing work to monitor the bacteria associated with soft rot disease.

## Figures and Tables

**Table 1:** Pectinolytic isolates, their genetic identities, and phenotypic characteristics.

Isolate	Isolation Host	Genus/Species	Pathogenic to Potato	Pathogenic to Chinese (Napa) Cabbage)	Swim	Swarm	AHL Production	Lactose	Protease
M1	Onion	not determined	−	−	+	+	−	+	−
M2	Lettuce	*Pseudomonas* sp.	−	−	+	+	−	−	+
M3	Lettuce	*Pectobacterium carotovorum*	+	+	+	+	+	+	+
M4a	Lettuce	*Pectobacterium carotovorum*	+	−	+	+	+	+	+
M4b	Lettuce	not determined	−	−	+	+	−	−	+
M5	Lettuce	*Chryseobacterium indoltheticum*	+	−	+	+	−	+	+
M6	Lettuce	*Pseudomonas cedrina*	−	−	+	+	−	−	+
M7	Cilantro	*Pectobacterium carotovorum*	+	−	+	+	+	+	+
M8a	Cilantro	*Pectobacterium carotovorum*	+	−	+	+	+	+	+
M8b	Cilantro	*Pectobacterium carotovorum*	+	−	+	+	+	+	+
M9	Cilantro	*Lelliottia amnigena*	−	−	+	+	−	−	+
M10	Cilantro	*Pectobacterium carotovorum*	+	+	+	+	+	+	−
M11	Spinach	*Pseudomonas azotoformans*	−	−	+	+	−	−	+
M12a	Zucchini	*Pseudomonas allopoutida*	−	−	+	+	−	−	−
M12b	Zucchini	*Escherichia fergusonii*	−	−	+	+	−	−	−
M13	Onion	*Pseudomonas marginalis*	+	−	+	+	−	−	+
M14	Onion	*Pseudomonas petroselini*	−	−	+	+	−	+	+
M15	Potato	*Stenotrophomonas maltophila*	−	−	+	+	−	−	+
M16	Potato	not determined	−	−	+	+	−	+	+
M17	Spinach	*Achromobacter spanius*	−	−	+	+	−	−	+
M18	Spinach	*Pseudomonas cyclaminis*	+	−	+	+	−	−	+
M19	Spinach	*Stenotrophomonas rhizophila*	−	−	+	+	−	−	+
M20	Spinach	*Pseudomonas* sp.	+	−	+	+	−	−	+
M21	Celery	*Pseudomonas composti*	−	−	+	+	+	+	+
M22a	Celery	*Stenotrophomonas maltophila*	−	+	+	+	−	−	+
M22b	Celery	*Pectobacterium carotovorum*	+	−	+	+	+	+	+
M23	Carrot	*Pseudomonas grimontii*	−	−	+	+	−	−	+
M24	Carrot	*Pantoea* sp.	+	−	+	+	+	−	+

+ represents a positive reaction for each phenotype, and − represents a negative reaction for the phenotype.

**Table 2:** Sequencing data for isolates selected for genome sequencing.

Isolate	Species	Genome Size	Sequencing Coverage	Contigs	Putative Plasmids	Accession
M3	*Pectobacterium carotovorum*	4.80 Mb	103×	1	0	GCA_045038015.1
M4a	*Pectobacterium carotovorum*	4.79 Mb	46×	1	0	GCA_045038025.1
M5	*Chryseobacterium scophthalmum*	4.57 Mb	42×	1	0	GCA_045037995.1
M8a	*Pectobacterium carotovorum*	4.80 Mb	96×	1	0	GCA_045038005.1
M8b	*Pectobacterium carotovorum*	4.80 Mb	102×	1	0	GCA_045037985.1
M10	*Pectobacterium versatile*	4.88 Mb	101×	1	0	GCA_045037945.1
M12a	*Pseudomonas alloputida*	6.27 Mb	46×	4	3	GCA_045037975.1
M13	*Pseudomonas marginalis*	6.70 Mb	85×	4	3	GCA_045037965.1
M14	*Pseudomonas petroselini*	6.93 Mb	57×	2	1	GCA_045037875.1
M20	*Pseudomonas* spp. within *P. koreensis* group	6.76 Mb	62×	2	1	GCA_045037865.1
M22b	*Pectobacterium versatile*	5.14 Mb	100×	1	0	GCA_045037855.1

## Data Availability

All genome sequences generated as part of this study are available through NCBI under BioProject number PRJNA1183984.

## Supplementary Materials

The following supporting information can be downloaded at: https://www.mdpi.com/article/10.3390/pathogens13121096/s1, Table S1: Produce samples collected and tested, Table S2: Top BLAST hits for 16S sequences, Table S3: Average nucleotide identity of *Pectobacterium* and *Dickeya* species, Table S4: Average nucleotide identity of *Pectobacterium carotovorum*, Table S5: Average nucleotide identity of *Pectobacterium versatile*, Table S6: Average nucleotide identity of *Pseudomonas* species, Table S7: Average nucleotide identity of *Chryseobacterium* species, Table S8: Sequences of predicted prophage elements in *Pectobacterium* genomes, Table S9: Top BLAST hits of prophages using all *Pectobacterium* genomes in NCBI; Figure S1: Representative CVP plate from isolation of pectinolytic bacteria, Figure S2: Potato slices inoculated with pectinolytic isolates, Figure S3: Chinese (napa) cabbage leaves inoculated with pectinolytic isolates.
