# Supplementary material for: Characterization and Genomics of Pectinolytic Bacteria Isolated from Soft Rot Symptomatic Produce
Source: Pathogens. 2024 Dec 12;13(12):1096. doi: 10.3390/pathogens13121096 (PMC11728799; doi:10.3390/pathogens13121096)
Supplement: Supplementary file 1 [file pathogens-13-01096-s001.zip › supplementalFigures.pdf]

Supplemental Figures for:

Characterization and genomics of pectinolytic bacteria isolated from soft rot symptomatic produce

Kyla Radke, Brandon Rivers, Mya Simpkins, Jacob Hardy, Jeffrey K. Schachterle

Figure S1	p2
Figure S2	p3
Figure S3	p4

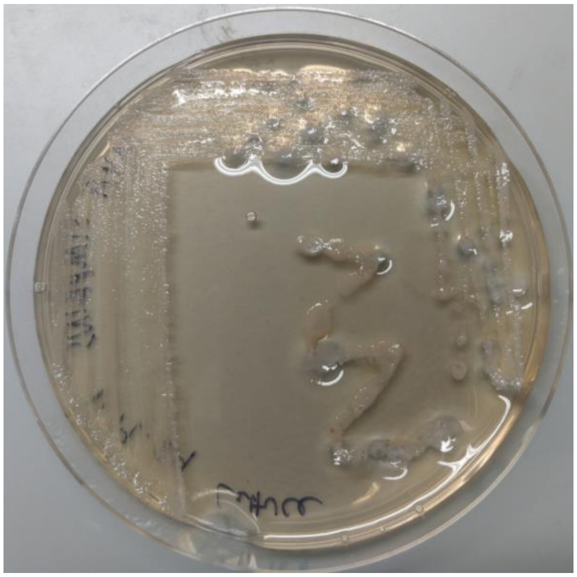

Figure S1: Representative CVP plate from isolation of pectinolytic bacteria. An abundance of growth is observed, with only select colonies causing pitting on the CVP media. Bacteria from pits were re-inoculated to CVP media until pure cultures were obtained.

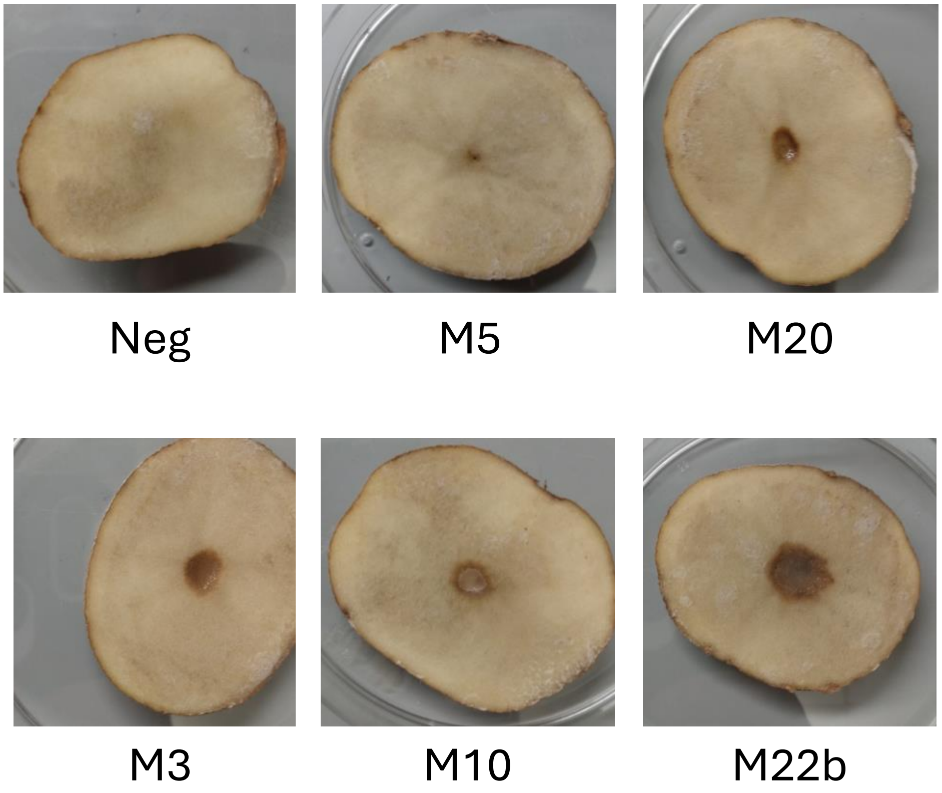

Figure S2: Potato slices inoculated with pectinolytic isolates. Slices incubated at 30°C for 20h post-inoculation. Representative images shown.

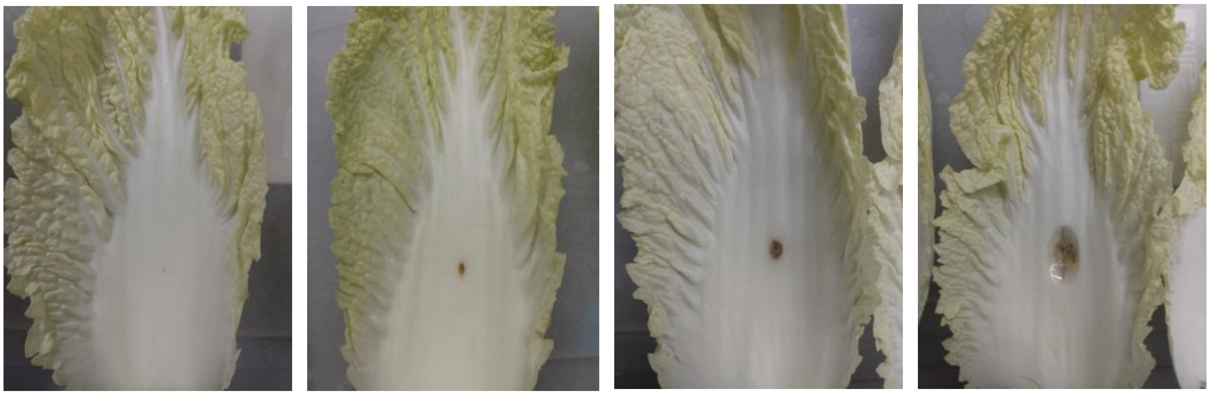

Figure S3: Chinese (napa) cabbage leaves inoculated with pectinolytic isolates. Leaves incubated at 30°C for 20h post-inoculation. Representative images shown.
